# In vivo measurement of three-dimensional load exerted on dental implants: a literature review

**DOI:** 10.1186/s40729-022-00454-y

**Published:** 2022-11-14

**Authors:** Itt Assoratgoon, Nobuhiro Yoda, Maya Iwamoto, Tomoya Sato, Tetsuo Kawata, Hiroshi Egusa, Keiichi Sasaki

**Affiliations:** 1 Division of Advanced Prosthetic Dentistry, Tohoku University Graduate School of Dentistry, 4-1, Seiryo-Machi, Sendai, Miyagi 980-8575 Japan; 2 Faculty of Dentistry, Chulalongkorn University, 34 Thanon Henri Dunant Wang Mai, Pathum Wan District, Bangkok, 10330 Thailand; 3 Department of Prosthodontics, Faculty of Dental Medicine, Universitas Airlangga, Surabaya, 60132 Indonesia; 4 Division of Molecular and Regenerative Prosthodontics, Tohoku University Graduate School of Dentistry, Sendai, Miyagi Japan

**Keywords:** Dental implant, Occlusal force, Measurement, In vivo, Load

## Abstract

**Background:**

For biomechanical consideration of dental implants, an understanding of the three-dimensional (3D) load exerted on the implant is essential, but little information is available on the in vivo load, including the measuring devices.

**Purpose:**

This review aimed to evaluate studies that used specific load-measuring devices that could be mounted on an implant to measure the functional load in vivo.

**Materials and methods:**

An electronic search utilizing the internet research databases PubMed, Google Scholar, and Scopus was performed. The articles were chosen by two authors based on the inclusion and exclusion criteria.

**Results:**

In all, 132 studies were selected from the database search, and 16 were selected from a manual search. Twenty-three studies were finally included in this review after a complete full-text evaluation. Eleven studies were related to the force measurements using the strain gauges, and 12 were related to the piezoelectric force transducer. The principles of the two types of devices were completely different, but the devices produced comparable outcomes. The dynamics of the load magnitude and direction on the implant during function were clarified, although the number of participants in each study was small.

**Conclusions:**

The load exerted on the implant during function was precisely measured in vivo using specific measuring devices, such as strain gauges or piezoelectric force transducers. The in vivo load data enable us to determine the actual biomechanical status in more detail, which might be useful for optimization of the implant prosthetic design and development of related materials. Due to the limited data and difficulty of in vivo measurements, the development of a new, simpler force measurement device and method might be necessary.

**Supplementary Information:**

The online version contains supplementary material available at 10.1186/s40729-022-00454-y.

## Background

The excessive load exerted on an implant has been considered one of the primary factors causing peri-implant bone resorption, and the harmfulness of so-called “overload” has been discussed since the 1990s [1–3]. Thereafter, various studies reported that the load required for peri-implant resorption was beyond the level of normal occlusal force [4–6]. Therefore, a very low possibility of overload-related peri-implant bone resorption is generally presumed.

On the other hand, an appropriate magnitude of load is generally essential to maintain or increase bone density [7, 8]. Indeed, an important role of the functional load on the implant in the long-term health of the peri-implant bone has been suggested [4, 9]. However, bone remodelling activities might depend on the individual, location, and other anatomic and physiological parameters [10, 11]. In particular, some clinical cone beam computed tomography (CBCT) follow-up studies suggested that load-related peri-implant bone remodelling might be related to the original bone being thin [12, 13]. Therefore, the nature of the effects of load on the peri-implant bone still needs to be well understood to obtain the long-term health of peri-implant bone.

Namely, it is essential to know the actual load exerted on the implant during relevant activities to better elucidate the influence of load on peri-implant bone remodelling. Several devices have been used both clinically and experimentally to measure the actual load on the implant in vivo [14]. In most of those studies, the static occlusal force was measured using a sheet-type sensor covering full dentition (i.e., Dental Prescale (GC, Tokyo, Japan)) [15] or a biting-type sensor covering a limited area in the dentition (i.e., GM10 occlusal force-meter (Nagano Keiki, Tokyo, Japan)) [16] by engaging those devices between the upper and lower dentition. However, those methods can only measure the load magnitude at one time fragment during clenching when biting. Of note, it is more important to know the 3-dimensional (3D) real-time load exerted on the implant during functions, such as chewing, under natural occlusal conditions.

A specific high-precision small load-measuring device must be installed into the structure of implant-supported prosthetic devices to measure the load on the implant during various oral functions. Furthermore, the measuring device itself must be in a form that does not impair oral function so that the functional load on the implant can be measured in real time. Normally, engineering expertise is indispensable for developing such high-precision devices. Therefore, load measurements on implants in vivo have been very challenging, and few studies have been carried out. This study reviewed the articles that performed in vivo measurements of the load imposed on implants using specific devices and summarized the measured load data and clinical findings; these data will be useful in clinical practice and related biomechanical and mechanobiological research.

## Methods

### Literature search strategy

An electronic search was performed utilizing the internet research databases “PubMed”, “Google Scholar”, and “Scopus”. The search terms were constructed from the following terms: ((Dental implant) OR (Abutment)) AND ((Bite force) OR (Occlusal force) OR (Loading) OR (Mastication)) AND (In vivo) AND (Measurement)). A manual search was performed in addition to these database searches by checking the bibliography of all identified articles for potentially relevant additional studies. The search and selection process were concluded on 14 March 2022.

### Inclusion and exclusion criteria

The inclusion criteria established that the studies must record an in vivo load measurement and involve the usage of a force measuring device designed to measure force on each implant prosthesis. Additionally, the full text of the studies was required to be available in English. Studies were excluded from this review if they were in vitro or animal studies and used the methodology involving the external biting-type load-measuring devices, or the full text was not available.

### Study selection

**Fig. 1:**
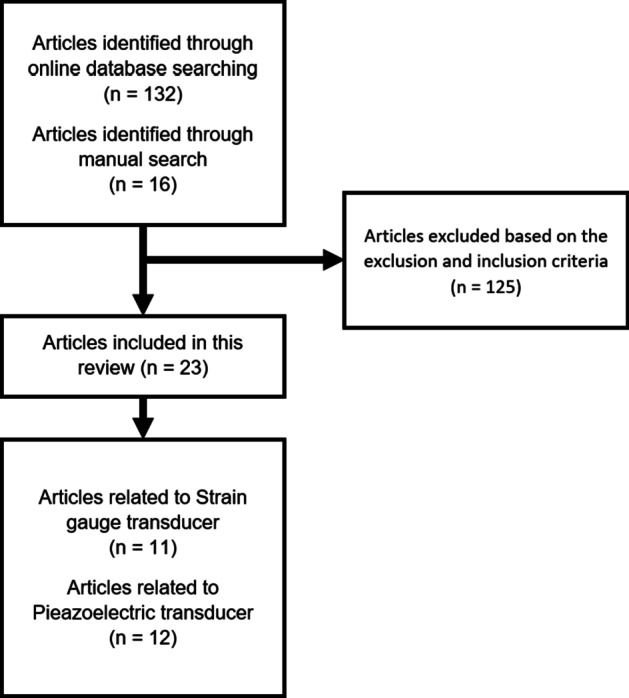
Chart of the article selection process

Figure 1 demonstrates the literature search strategy used in this study. Two authors (IA and NY) evaluated the literature search results. First, the collected titles and abstracts were selected according to the aim and predetermined criteria. Second, two authors confirmed the concurrence of the results, and the full text of these articles was read to further examine the details of the results reported. Subsequently, the discrepancies in the results from the two authors were discussed with other authors (MI and TS). Finally, the studies that satisfied the inclusion criteria were included.

## Results

### Search results

A total of 132 studies were matched with the search terms, and 16 studies were selected from the manual hand search results by two authors (IA and NY). The abstracts and titles of all articles were screened. Unrelated titles and articles with nonclinical force measurement experimental methodology were excluded.

Finally, 23 studies were selected (by IA and NY) for inclusion in the present review. Any discrepancies in the results from the two authors were discussed with other authors (MI and TS). It was found that either strain gauges or a piezoelectric force transducer was utilized as a tool to measure the load on the implant in all selected studies. Eleven studies were related to force measurements using strain gauges, and 12 were related to measurements using the piezoelectric force transducer. Studies not included (*n* = 125) in the review after reading the full texts and reasons for exclusion are listed in Additional file 1: Table S1.

### *Studies of *in vivo* force measurement using a strain gauge-based device*

The details of the load magnitude and moments demonstrated in each study are summarized in Table 1. The strain gauge-based load-measuring system was used to measure the axial load and bending moment exerted on the implant and/or surrounding structures during clenching or mastication in humans.

**Table 1:** Studies of in vivo force measurements using a strain gauge-based device

Author	Number of participants/implants	Force measurement devices		Implant location	Types of prosthesis	Task	Measured load range (direction (moment))	Load magnitude (values)	Main findings
		Type of strain gauge	Number of gauges per implant						
Hobkirk et al. [17]	5/5	Type 3/120/PC11, (Tinsley & Co Ltd., Croydon, England)	2	Mandible	Single crown	MVC, chewing	V	Acrylic teeth - Nuts 30.6N - Carrots 42.8N - Bread 35.2N - Tapping 9.8N Porcelain teeth - Nuts 35N - Carrots 30.2N - Bread 35N - Tapping 11.4N	Mean peak masticatory forces varied considerably from subject to subject but were consistent within each subject No differences were detected in the load rates associated with the use of porcelain or acrylic occlusal material
Glantz et al. [18]	1/1	Type EA-06-015EH-120; (Micromeasurements, Romulus, MI, USA)	4	Mandible	Fixed partial denture	MVC, chewing	V, L, AP, A Bending moment (Approximately 18 Ncm when chewing bread, as shown in the charts)	Not described (Approximately 15–20N of axial force shown in charts)	A difference in biting force was observed in in vivo and in vitro experiments
Gunne et al. [19]	5/10	Type EA-06-015EH-120; Micromeasurements, Romulus, MI, USA	3	Posterior mandible	Fixed partial denture	MVC, chewing	V	Mean chewing force on the Mesial implant 24N Distal implant 11N Connected implant 16N	The functional load on one implant support and one natural tooth support does not differ from that of two-implant support
Richter et al. [20]	10/10	Type LY 11, Hottinger Baldwin Meßtechnik, Darmstadt, Germany	1	Molar and premolar	Single crown	MVC	V	Max. force 120–150N	Shock-absorbing intramobile elements in implants may not be necessary
Richter et al. [21]	9/18	Type LY 11, Hottinger Baldwin Meßtechnik, Darmstadt, Germany	2	Molar	Single crown	Chewing	V, L, AP	Transverse loading: Max. 170 Nmm Clenching: Max. 140 Nmm	During functions, transverse forces bend the implant in a buccolingual direction that causes the highest interface stress
Duyck et al. [22]	11/35	Type FLG-02–11 of TML; Tokyo Sokki Kenkyujo Co., Ltd	3	Mandible and maxilla	Two- or three-unit fixed partial prostheses	50N controlled vertical force	V, L, AP	Not described (shown in charts)	A significantly better distribution of bending moments with the metal prostheses. No difference in load distribution with the different prosthesis materials
Duyck et al [23]	13/76	Type FLG-02–11 of TML; Tokyo Sokki Kenkyujo Co., Ltd	3	Mandible and maxilla	Fixed full prosthesis	50N controlled vertical force	L, AP	Not described (shown in charts)	Distal extensions exert more compressive force on the closest implant. Higher forces were observed with a decreasing number of supporting implants
Bassit et al. [24]	5/5	Type EA-06-015EH-120; Micromeasurements, Romulus, MI, USA	3	Premolar	Single crown	Biting, chewing	V	Acrylic resin patient 1 198N patient 2 154N patient 3 450N patient 4 2055N patient 5 995N Ceramic patient 1 195N patient 2 63N patient 3 96N patient 4 1039N patient 5 1280N	The different occlusal materials did not lead to different forces on the implants of the patients
Morneburg et al. [25]	9/18	Type FAE-02 W-35-S6, BLH	4	Molar and premolar	Three-unit fixed partial prostheses	Chewing	V	Posterier 314N max Anterior 91N max	Measuring chewing force by bending the pontic is sensitive to the site of force and involves a risk of underestimation
Morneburg et al. [26]	10/20	Type FAE-02 W-35-S6, BLH	4	Molar and premolar	Three-unit fixed partial prostheses	Chewing	V, L,AP	Mean vertical force 264–284N	Narrowing the occlusal surface reduces lateral force loading
Kim et al. [27]	3/6	Type EA-06-031DE-120; Micro Measurements Division, Wendell, NC, USA	2	Mandible	Three-unit fixed partial prostheses	MVC	V	patient 1 (Rt) -Gold 231N -Porcelain 252N -Tescera 243N patient 1 (Lt) -Gold 62N -Porcelain 114N -Tescera 50N patient 2 -Gold 51N -Porcelain 52N -Tescera 68N patient 3 -Gold 118N -Porcelain 138N -Tescera 68N	Tescera ATL, porcelain fused to metal, can function as an occlusal material for implant treatment

*V* vertical, *L* lateral, *AP* anteroposterior, *A* measured all directions simultaneously, *MVC* maximum voluntary clenching

Basically, multiple strain gauge units need to be adhered onto an implant abutment surface to acquire an accurate load pattern. Hobkirk et al. used 2 units of strain gauges on each implant and reported that the load on the implant depended on the superstructure materials [17].

In several studies, 4 units of strain gauges were placed perpendicular to each other on their long axes and positioned 90 degrees to each other [18, 25, 26]. However, some studies have used 3 strain gauges placed 120 degrees from each other [19, 22, 23]. Richter et al. only used 1 unit [20], while their subsequent study and other researchers used 2 units of strain gauges on each implant [21]. Duyck et al. [22] applied the 3 strain gauge-based load-measuring system to multiple implants supporting fixed partial dentures to measure the load distribution and bending moments. The authors clarified the influence of the superstructure materials on the force distribution. A software program for analysing the load that makes the device user-friendly and simple was also updated [23]. They revealed that the distal extensions exerted greater compressive force on the closest implant, and higher forces were observed with a decreasing number of supporting implants.

Another experiment [24] in which 3 strain gauges were attached on the abutment in a single implant-supported prosthesis revealed the load magnitude during clenching and 3D chewing motions. The authors clarified that the different occlusal materials did not lead to different forces generated on the implants and the load on the implants varied across the patients, and surprisingly over 2000 N was detected in a certain patient. Another group measured the strain of the superstructure using strain gauges placed under the pontic in the case of a bride type of implant-supported 3-unit fixed partial denture, and an underestimated larger force magnitude was observed with this approach compared to that obtained with gauges attached directly to the abutment [25]. The measured load mainly depended on the location of the force application site and greater bending occurred when the force was applied at the centre of pontic compared to applying it near the abutment. Hence, a reliable measurement with the pontic method would require that a load is always applied above the strain gauge, which might result in more or less misleading force values when the loading position changes, such as when chewing foods. The method in which the strain gauges are attached to the abutment might be a more suitable application for recording measurements during natural mastication. The same group [26] analysed the effect of the occlusal surface condition of fixed prostheses, namely, the cusp inclination, on the masticatory force exerted on the supporting implants. The load exerted on the implant was also measured and compared among different materials of superstructures using similar devices with strain gauges [27]. The authors described that no significant differences in normalized bending moments were observed among the different materials; however, the load on the implants differed significantly across the patients.

### *Studies of *in vivo* force measurement using a piezoelectric transducer-based device*

The details of the load magnitude and directions demonstrated in each study are summarized in Table 2. For the application of the small type of piezoelectric transducer, which was involved in the stainless-steel housing, into the dental implant, to the best of our knowledge, only two research groups have been used for the measurement of the load on the implant in humans. Mericske-Stern et al. [28] from Bern University developed an in vivo measurement system for the load on implants with piezoelectric transducers (Kistler Instruments, Winterthur, Switzerland). The device was mounted directly on the tissue-level implant (Standard RN, Straumann, Basel, Switzerland). This transducer enabled us to complete a series of studies based on in vivo measurement of the load exerted on the implants supporting an overdenture. The measured forces on the implant supporting a complete overdenture during clenching or chewing were greater than those measured during light tapping or grinding [28]. The authors also investigated the effect of distal bar extensions on the load on the implants and revealed that the distal bar extension reduced the load-sharing effect of bars [29]. They also clarified that the use of retentive ball anchorage reduced the load distributed to the implants compared to other attachments. In addition, chewing function resulted in a more pronounced transverse force component, particularly in the anterior direction, which exceeded the vertical force [30, 31]. A point of consideration was raised in that the preload of mounting the transducer on the implant could affect the reliability of the measurement. With a decrease in preload from its optimal value, the magnitude measured may be slightly reduced [32]. The authors concluded that a preload of ≥ 300N should be achieved in in vivo studies.

**Table 2:** Studies of in vivo force measurements using a piezoelectric transducer-based device

Author	Number of participants/implants	Device	Implant position	Prosthesis	Function	Load magnitude (values)	Main findings
Mericske-Stern et al. [28]	5/10	Kistler Piezo-Instrumentation, Winterthur, Switzerland	Mandible	Removable full denture	Maximum occlusal force, chewing, light tapping, grinding	Maximal Occlusal Force: z-axis 18 to 240N; y-axis 8 to 50N; x-axis 8 to 110N Chewing Test Food: z-axis 1.5 to 260N; y-axis 1.5 to 66N; x-axis 5 to 62N Light Tapping and Grinding: z-axis 1 to 99N; y-axis 1 to 50N; x-axis 1 to 28N	The maximum occlusal force is lower than that of natural teeth The chewing force consists of a vertical force and a smaller lateral force
Mericske-Stern et al. [29]	5/10	Kistler Piezo-Instrumentation, Winterthur, Switzerland	Mandible	Removable full denture	Maximum biting force in centric, parafunction, chewing	Not described (shown in charts)	Distal bar extension reduces the load-sharing effect of bars
Mericske-Stern et al. [30]	5/10	Type Z15657, Kistler Instruments AG, Winterthur, Switzerland	Mandible	Removable full denture	Maximum biting force in centric, biting on a bite plate, grinding, chewing	Not described (shown in charts) 0–150N in the z-axis	The use of retentive ball anchorage reduces the force distributed to the implants Chewing function resulted in a more pronounced transverse force component, particularly in the anterior direction, which exceeded the vertical force magnitudes (in the ball attachment)
Mericske-Stern et al. [31]	5/10	Kistler Instrumente AG, Winterthur, Switzerland	Maxilla	Removable full denture	Maximum biting force, biting on a bite plate, chewing	not described (shown in charts)	Load sharing of overdenture and fixed complete denture is not significantly different
Yoda et al. [33] Shigemitsu et al. [34]	1/2	Type Z18400, Kistler Instrument, Winterthur, Switzerland	Mandible (45,46)	Fixed partial denture	MVC, biting paraffin wax	Results of MVC Splinted model 45: 100.1N 46: 111.4N Non-Splinted model 45: 34.5N 46: 176.9N	The load on the implants was more distributed in the case of splinted superstructure
Shigemitsu et al. [35], Sato et al. [36]	1/2	Type Z18400, Kistler Instrument, Winterthur, Switzerland	Mandible	Removable full denture (2 or 4 implant-supported)	MVC	4 implant-supported overdenture Implant 1: 44.0 (right side) Implant 2: 41.5 Implant 3: 43.5 Implant 4: 63.8 (left side)	The total load in the 4IOD was larger than that of the 2IOD The load on each implant in the 2IOD was larger than that of the 4IOD
Kobari et al. [37]	1/3	Type Z18400, Kistler Instrument, Winterthur, Switzerland	Mandibular molar and premolar	Three-unit fixed partial prostheses	Gum chewing (10 chewing cycles)	Load magnitudes (35, 36, and 37) (Newtons) MVC 3 implant-supported: (59.0, 70.7, and 61.8), Bridge: (114.4, NA, and 71.4), Distal cantilever type: (74.4, 111.8, and NA), Mesial cantilever type: (NA, 216.5, and 37.7) Gum chewing (10 cycles) 3 implant-supported: (79.2, 88.1, and 124.0), Bridge: (138.8, NA, and 172.3), Distal cantilever type: (33.9, 318.9, and NA), Mesial cantilever type: (NA, 277.6, and 66.5)	The load on implants in 3-implant supported and bridge cases was more distributed than that in cantilevered bridge cases The load direction changed dynamically when chewing
Yoda et al. [38] Zhang et al. [39]	2/2	Type Z18400, Kistler Instrument, Winterthur, Switzerland	Mandibular molar and premolar	Single crown	MVC, Gum chewing, Peanut chewing	MVC results patient 1: 38.50 ± 3.91N/patient 2: 115.94 ± 6.02N Peanut chewing: patient 1: 83.78 ± 24.5N/patient 2: 101.38 ± 14.02N Gun chewing: patient 1: 45.90 ± 8.17N/patient 2: 71.99 ± 9.69N	The load magnitude during MVC is lower than that during chewing The load magnitude is affected by food texture
Bing et al. [40]	1/1	Type Z18400, Kistler Instrument, Winterthur, Switzerland	Mandibular molar and premolar	Single crown	MVC, tapping, grinding	MVC: 177.6N Grinding: 46.5N Tapping: 32.4N	The functional load affects the stress distribution in the implant Stress in the implant increases with an increase in bone resorption

Measured load range are all in 3D, *MVC* maximum voluntary clenching

Another group used a piezoelectric force transducer (Kistler Instrument, Winterthur, Switzerland) with the in vivo load measurement system from Tohoku University in Japan. The authors [33, 34] investigated the load distribution between 2 implants supporting a fixed partial denture and revealed the effects of splinting the superstructure on the load distribution to both implants. Shigemitsu et al. [35, 36] used the same transducers and revealed that the load on each implant was smaller in a 4 implant-supported mandibular complete overdenture than that in 2 implant-supported type. Kobari et al. [37] applied this system to a 3-unit implant-supported fixed partial denture and investigated the effects of different numbers and configurations of supporting implants on the load distribution. The authors clarified the dynamic changes in the 3-dimensional load magnitude and direction during relevant functions in real time and the load on the implant of the cantilevered bridge type was significantly larger than the conventional bridge type. The authors also clarified that the load on the implant at gum chewing was basically larger than that at maximum voluntary clenching (MVC). Other studies [38, 39] reported that the load magnitude was affected by food texture, and the load magnitude during chewing was larger than that during MVC. The data were subsequently used for advanced experimental and numerical purposes, such as finite element analysis (FEA), and the effect of peri-implant bone resorption on the stress distribution in implant body was investigated [40].

## Discussion

Among various studies regarding in vivo measurement of load on implants, this review paper focused on those in which the load was measured during function, such as clenching and mastication, in a natural occlusal condition by incorporating a specific sensing device into the implant components or superstructure. Due to their suitable size, high accuracy, wide measuring range, and high adaptability, both strain gauge-based devices and piezoelectric force transducers have been utilized for such measurements thus far.

For the measurement using the strain gauges, multiple strain gauge units are needed to acquire an accurate load pattern. Glantz et al. [41] used the Rosette strain gauge, a formation of 3 strain gauges in various orientations, to measure force on a dental bridge in multiple directions. Brunski et al. [42] developed an epoch-making load-measuring system consisting of a custom-made abutment with 3 strain gauges attached. This system was further used to quantify the amount of load applied to the implant by measuring the amount of distortion of the abutment under functional load in prosthesis-related experiments [43]. On the other hand, the piezoelectric force transducer involves some piezoelectric element materials that generate an electric charge when the load is applied. The charge is then amplified by a specific amplifier in the circuit and translated into an output voltage proportional to the load exerted on the piezoelectric crystal. The piezoelectric force transducer was first introduced for use as a bite force measuring device in the dental field by Graf et al. [44]. By installing the transducer including 3 piezoelectric elements in a fixed partial bridge, 3-dimensional forces applied to the bridge were measured. Although the principles of both methods are completely different, the outcomes are comparable to each other, and an ongoing debate about which method is superior remains. Considering the real-time accurate sensing and fast responsiveness, the piezoelectric force transducer might be more suitable for measuring the functional load on the implant in vivo.

Load data measured in vivo that quantitatively demonstrated the changes in the load magnitude and direction in real time enabled us to know the actual biomechanical status during oral function in more detail. In the 3-unit implant-supported fixed prosthesis, for example, a remarkably large load, greater than 600 N, was exerted on the implant adjacent to the pontic when using the cantilevered bridge-type superstructure during gum chewing, although the patient was an elderly woman [37]. Ducky et al. also described the increased load on the closest implant to the cantilever extension [23]. Unlike the static load to the implant during clenching, dynamic loads from various directions were also found to be exerted on the implant during chewing. The dynamic changes in the magnitude and direction of the load on the implant might be due to not only the food properties and texture [38] but also to the control mechanism and the configuration of the masticatory components [45, 46]. Three factors affecting the load directional change during functions were suggested: the friction between the two contacting teeth, the contact sliding along the incline planes of the cusp, and the formation of new contacts and loss of existing contacts influenced by the periodontal ligament. However, these findings and suggestions were obtained from a simulation study [47]. The load exerted during bruxism might be an unexpectedly large load, thus causing some mechanical complications [48], and the magnitude of intraosseous stress during bruxism might destroy osseointegration [49, 50]. Accumulation of load data measured in vivo is essential, and in particular, the detailed load data during such parafunctional habits should be valuable not only for the prevention of various biomechanical and biological complications regarding the osseointegrated implant, but also for optimization of the implant prosthetic design and related material development.

On the other hand, investigating the behaviour of the load on the implant compared to that on the natural tooth with the periodontal ligament is also important. Bassit et al. [24] revealed that a difference in resilience between acrylic resin and ceramic veneering materials used as the implant superstructure, but that difference was only measurable in in vitro where the force was generated by a shock only and the implant was rigidly anchored. The authors considered that the resilience of the masticatory system was overriding the difference in the stiffness of the various materials. The resilience in the periodontal ligament of any antagonist natural tooth might also be affected. The load on the tooth is controlled by a physiological neuromuscular mechanism, such as the muscle spindle and periodontal-masseteric reflex, during mastication [51, 52]. However, mastication is normally carried out smoothly on implant prostheses, despite the lack of periodontal ligaments, which suggests some adaptive mechanisms related to tooth loss and replacement with implants, such as activation of periodontal ligament function of opposing teeth, elicitation of neuroplasticity, and activation of osseoperception [47, 53–55]. In vivo measured load data will greatly contribute to the elucidation of these mechanisms.

The measured force on the implant may be affected by not only the tasks but also individual factors, such as facial morphology or masticatory muscle force. In vivo load measurement will be valuable to understand the effects of individual factors on the loads. Considering the large individual differences in human bite force [56], the wide range of measured force magnitudes among the patients (63–2055N [24]) might be reasonable. However, no studies used the strain-gauge system described the output calibration of the measuring device for that range. On the other hand, in the studies using the piezoelectric transducers, the load-measuring device was calibrated for the specified measurement range [32, 57]; thus, the measured load data might be more reliable. Clearly, the implant load changed not only vertically but also three-dimensionally during mastication and clenching [37]. In particular, for the cantilever configuration, the load increased in both the vertical and lateral directions. This excessive load might cause some mechanical complications, such as screw loosening and fracture. Therefore, we must consider the implant configuration from a biomechanical perspective in patients with multiple implants.

To date, the durability of the implant components against occlusal load and force-related peri-implant bone changes have been verified in numerous experimental studies [58]. Most of those experiments used fairly simplified loading conditions, i.e., static loading or cyclic loading tests to the implant due to the limited data of the load measured in vivo, which can be used as a reference. This might have been a general limitation of those experimental studies. To obtain more realistic results in in vitro or in silico simulation studies, reproducing the actual in vivo loading conditions as shown in this review as much as possible should be effective.

Some studies utilized in vivo measured load data for in silico simulation studies. Bing et al. [40] reported an increased risk of implant fracture associated with the amount of bone resorption due to peri-implantitis using an FEA of in vivo measured loads. The in vivo load data have also already been evaluated using FEA to investigate the distribution of peri-implant bone in a stimulation [34–36]. Indeed, the reliability of the simulation result was substantially improved using the in vivo measured value. Furthermore, those FEAs were utilized to construct algorithms analysing peri-implant bone remodelling [59, 60]. By comparing the results from the FEA with actual clinical outcomes of peri-implant bone changes measured in computed tomography (CT) images over time, the patient-specific bone remodelling algorithm was constructed [60]. The patient-specific bone remodelling algorithm can be extremely important data for the basis of personalized medicine in implant treatment.

A common limitation of the in vivo measurement experiments in any study introduced in this review was the small number of human participants. This is thought to be due to the cost of preparing each load-measuring device. Additionally, mounting the device to the implant components or superstructure for measuring the load under the natural occlusal condition, unlike the method of just biting the device, such as the Dental prescale, takes a large amount of time and requires great effort. Elaborate parts and precise work are essential for accurate measurement devices, which are challenging to prepare in large numbers. Difficulty securing participants was also expected. New measurement methods that replace the previous ones using strain gauges or piezoelectric transducers introduced in this review will thus be needed. We were unable to statistically analyse the loading status on implant because of the high degree of heterogeneity in load-measuring methods and the tasks performed during the measurement. Furthermore, the load-measuring devices introduced in this review are incapable of measuring the stress distribution not only in the implant components but also in the peri-implant bone as an important effect of the loading on living organisms. However, as mentioned above, the actual in vivo load data should be useful for applications in computational aided engineering methods such as FEA for investigating those stress distributions.

## Conclusions

An understanding of the functional load exerted on dental implants is essential to obtain long-term favourable treatment outcomes. This review demonstrated that for the in vivo measurement of the load on implants, attaching strain gauges to the implant components and using piezoelectric force transducers have been the mainstream methods. Although both approaches have demonstrated the actual measured load on the implant during function, 3D quantitative load measurement could be carried out by using the method with a piezoelectric transducer. The development of new measurement methods is also expected to accumulate much more patient data.

## Supplementary Information

**Additional file 1: Table S1.** Studies not included (*n*=125) in the review after full text reading and reason for exclusion.

## Data Availability

The data sets generated and analysed during the current study are available from the corresponding author upon reasonable request.

## Abbreviations

CBCT: Cone beam computed tomography
3D: Three-dimensional
MVC: Maximum voluntary clenching
FEA: Finite element analysis
